# Supplementary material for: Personalized Metabolic Avatar: A Data Driven Model of Metabolism for Weight Variation Forecasting and Diet Plan Evaluation
Source: Nutrients. 2022 Aug 26;14(17):3520. doi: 10.3390/nu14173520 (PMC9460345; doi:10.3390/nu14173520)
Supplement: Supplementary file 1 [file nutrients-14-03520-s001.zip › nutrients-1860046-supplementary.pdf]

nutrients MDPI

Supplementary materials

Personalized Metabolic Avatar: A Data Driven Model of Metabolism for Weight Variation Forecasting and Diet Plan Evaluation

Alessio Abeltino [1,2], Giada Bianchetti[1,2], Cassandra Serantoni[1,2], Federico Ardito[3], Daniele Malta[3], Marco De Spirito[1,2] and Giuseppe Maulucci[1,2,*]

[1] Neuroscience Department, Biophysics Section, Università Cattolica del Sacro Cuore, 00168 Rome, Italy
[2] Fondazione Policlinico Universitario A. Gemelli IRCSS, 00168 Rome, Italy
[3] RAN Innovation, Viale della Piramide Cestia, 00153 Rome, Italy
* Correspondence: giuseppe.maulucci@unicatt.it; Tel.: +39-06-30154265

S1. Seasonality analysis

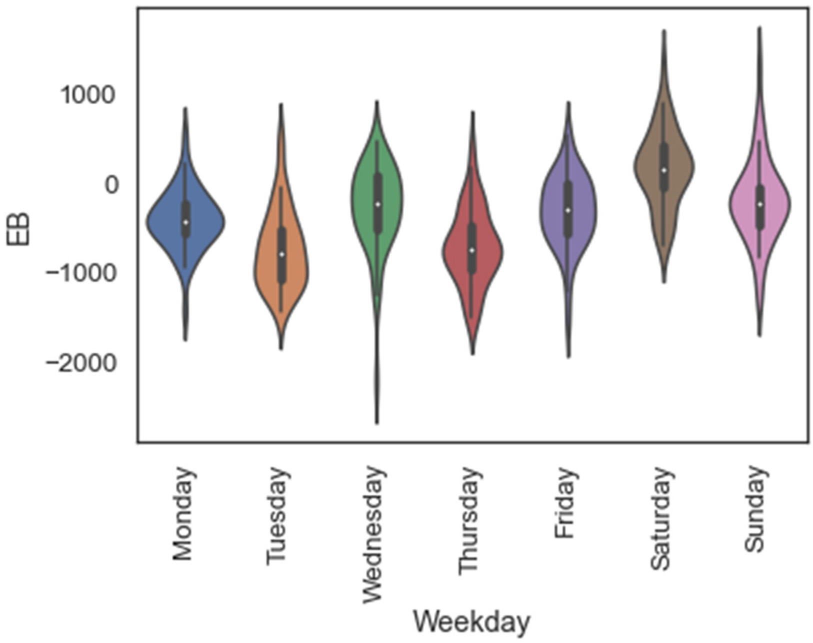

Figure S1. Violin plot of the EB for all days in a week.

For each user, an ANOVA test was carried out to check if a significant variation existed through the weekdays. The p values obtained from ANOVA analysis were significant ($p < 0.05$) for Users 1 and 2, and, therefore, we concluded that seasonal differences could exist (as an example, the results are shown in Table S1), and it was reasonable to include seasonal terms in the model.

Table S1: ANOVA Analysis Results.

	sum_sq	df	F	PR(>F)	EtaSq
**Weekday**	9.270996e+06	1.0	38.748621	1.580438e-09	0.112072
**Residual**	7.345283e+07	307.0	NaN	NaN	NaN

S2. *EB* Correction

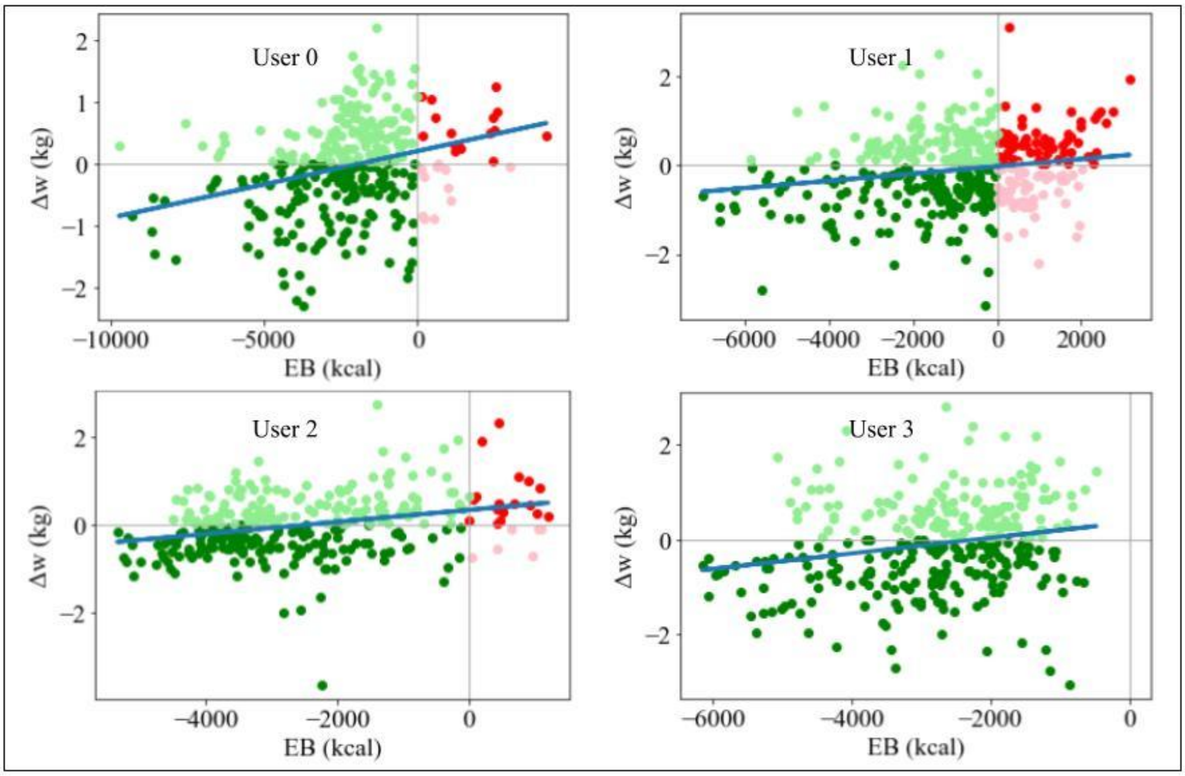

Figure S2. Distribution of weekly *w* versus weekly *EB* for all users.

S3. Theory of RNN: GRU and LSTM models

The basic RNN cell incorporates the dependence of the inputs by having a hidden state, or memory, that holds the essence of what has been observed so far. The value of the hidden state at any point in time is a function of its value at the previous time step and the value of the input at the current time step, and it is defined through the formula:

$$h_t = \phi(h_{t-1}, X_t), \quad \text{(S1)}$$

Here, ht and ht-1 are the values of the hidden states at time t and t-1, respectively, and Xt is the value of the input at time t (that in our case consists of the food's composition and the weight in preceding time steps). The recursiveness of formula (1) allows the RNN to be able to encode and incorporate information from arbitrarily long sequences.

As for traditional neural networks, the RNN's parameters are defined by three weight matrices U, V and W, corresponding to the weights of the input, output, and hidden states, respectively. These weight matrices are shared between each of the time steps because the same operation is applied to different inputs at each time step. This reduces the number of parameters that the RNN needs to learn.

We can describe the RNN in terms of the following equations:

$$h_t = tanh(Wh_{t-1} + Ux_t), \quad \text{(S2)}$$

$$y_t = softmax(Vh_t), \quad \text{(S3)}$$

The internal state of the RNN at time *t* is given by the value of the hidden vector $h(t)$, which is the sum of the weight matrix *W* and the hidden state h_{t-1} at time *t-1*, and the product of the weight matrix *U* and the input x_t at time *t*, passed through a *tanh* activation function. In our application, the input x_t corresponds to the following variable set: *w*, m_C ,m_L , m_P and *EB*. The choice of *tanh* over other activation functions such as *sigmoid* has to do with it being more efficient for learning in practice, and helps combat the vanishing gradient problem (2). The output vector

y_t at time *t* is the product of the weight matrix *V* and the hidden state h_t, passed through a *softmax* activation, such that the resulting vector is a set of output probabilities (3).

Like traditional neural networks, training RNNs also involves backpropagation of gradients. The only difference is that in this case, since the weights are shared by all time steps, the gradient at each output depends not only on the current time step, but also on the previous ones. This process is called backpropagation through time (BPTT) [1]. For that reason, we need to sum up the gradients across the various time steps in the case of BPTT. This is the key difference between traditional backpropagation and BPTT. During backpropagation, the gradients of the loss with respect to the weights are computed at each time step, and the parameters updated with the sum of the gradients.

A well known problem encountered when training artificial neural networks is the vanishing and exploding gradient problem. This problem manifests as the gradient of the loss (with respect to W matrix) approaching either 0 or infinity, making the network hard to train.

During each iteration of training, each weight of the neural network receives an update proportional to the partial derivative of the error function with respect to the current weight. The problem is that in some cases, the gradient will be vanishingly small, effectively preventing the weight from changing its value. In the worst case, this may completely stop the neural network from further training.

There are a few approaches to minimizing the problem, such as proper initialization of the W matrix, more aggressive regularization, using ReLU instead of tanh activation, and pre-training the layers using unsupervised methods. However, the most popular solution is to use LSTM or GRU architectures that have been designed to deal with this problem and learn long-term dependencies more effectively.

The LSTM is a variant of the SimpleRNN cell that is capable of learning long-term dependencies. It implements recurrence similarly to RNN cells, but instead of a single tanh layer, there are four layers interacting in a very specific way.

LSTM works around the vanishing gradient problem by using the gates i, f, o and g.

We can describe how the hidden state h_t at time *t* is calculated from the value of hidden state h_{t-1} at the previous time step using the following equations:

$$i = \sigma(W_i h_{t-1} + U_i x_t + V_i c_{t-1}), \tag{S4}$$

$$f = \sigma(W_f h_{t-1} + U_f x_t + V_f c_{t-1}), \tag{S5}$$

$$o = \sigma(W_o h_{t-1} + U_o x_t + V_o c_{t-1}), \tag{S6}$$

$$g = tanh(W_g h_{t-1} + U_g x_t), \tag{S7}$$

$$c_t = (f * c_{t-1}) + (g * i), \tag{S8}$$

$$h_t = tanh(c_t) * o, \tag{S9}$$

Here *i*, *f* and *o* are the *input*, *forget* and *output* gates. They are computed using the same equations but with different parameter matrices $W_i, U_i, V_i, W_f, U_f, V_f, W_o, U_o, V_o$. The sigmoid function modulates the output of these gates between 0 and 1, so the output vectors produced can be multiplied element-wise with another vector to define how much of the second vector can pass through the first one.

The *forget* gate defines how much of the previous state h_{t-1} you want to be allowed to pass through. The input gate defines how much of the newly computed state for the current input xt you want to let through, and the output gate defines how much of the internal state you want to expose to the next layer. The internal hidden state *g* is computed based on the current input x_t and the previous hidden state h_{t-1}.

Given *i*, *f*, *o* and *g*, the cell state *c* can be calculated at time *t* as the cell state c_{t-1} at time *t-1* multiplied by the value of the *forget* gate *f*, plus the state *g* multiplied by the *input* gate *i*. This is

basically a way to combine the previous memory and the new input. Finally, the hidden state h_t at time t is computed as the memory c_t at time t, with the *output* gate o.

LSTM is a drop-in replacement for a SimpleRNN cell; the only difference is that it is resistant to the vanishing gradient problem.

Instead, the GRU is a variant of the LSTM cell, capable of learning long-term dependencies.

It retains LSTM's resistance to the vanishing gradient problem; furthermore, its internal structure is simpler, and hence, it is faster to train since there are fewer computations needed to update the hidden state.

Instead of the i, f, and o gates in the LSTM cell, the GRU cell has 2 gates:

- z: update gate
- r: reset gate

The z gate is needed to define how much previous memory to keep around, while the r gate is needed to define how to combine the input with it.

The GRU cell defines the computation of the hidden state h_t at time t from the hidden state h_{t-1} at the previous time step using the following set of equations:

$$z = \sigma(W_z h_{t-1} + U_z x_t), \quad (S10)$$

$$r = \sigma(W_r h_{t-1} + U_r x_t), \quad (S11)$$

$$c = tanh(W_c(h_{t-1} * r) + U_c x_t), \quad (S12)$$

$$h_t = (z * c)((1 - z) * h_{t-1}). \quad (S13)$$

The outputs of the *update* gate z and the *reset* gate r are both computed using a combination of the previous hidden state h_{t-1} and the current input x_t. The sigmoid function modulates the output of these functions between 0 and 1. The cell state c is computed as a function of the output of the *reset* gate r and input x_t. Finally, the hidden state h_t at time t is computed as a function of the cell state c and the previous hidden state h_{t-1}. The parameters W_z, U_z, W_r, U_r and W_c, U_c are learned during training.

In conclusion, GRU neural networks are better than LSTM for our application because they are faster and thus more suitable for implementation in web-based applications, while keeping all of the LSTM advantages.

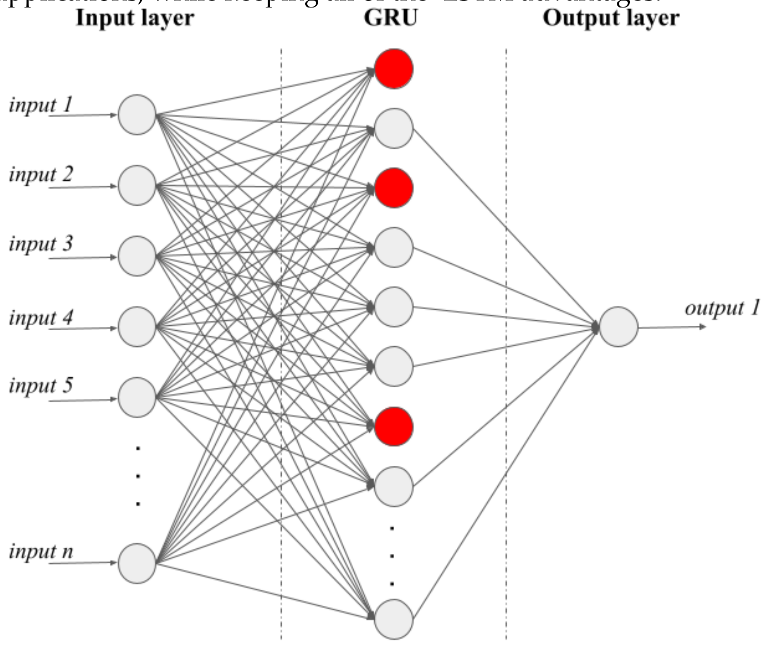

Figure S3. Distribution of weekly w versus weekly EB for all users.

S4. Regularization techniques

1. *Dropout*

Dropout is a regularization technique for reducing overfitting in neural networks by preventing complex co-adaptations on training data [2]. It is a very efficient way of performing model averaging with neural networks. It is a technique where randomly selected neurons are ignored during training. This means that their contribution to the activation of downstream neurons is temporarily removed on the forward pass and any weight updates are not applied to the neuron on the backward pass. If neurons are randomly dropped out of the network during training, others will have to step in and handle the representation required to make predictions for the missing ones. This is believed to result in multiple independent internal representations being learned by the network.

The effect is that the network becomes less sensitive to the specific weights of neurons, but in turn, it results in a network that is capable of better generalization and is less likely to overfit the training data. In tensorflow, the library used for the development of the model (https://www.tensorflow.org/api_docs/python/tf/keras/layers/Dropout, accessed on 7 July 2022), dropout is easily implemented by randomly selecting nodes to be dropped out with a given probability (e.g., 20%) in each weight update cycle. Dropout is only used during the training of a model and is not used when evaluating the skill of the model.

2. *Layer weight regularizers*

Regularization is the most used technique to penalize complex models in machine learning. It is deployed for reducing overfitting (or, contracting generalization errors) by making network weights small. Additionally, it enhances the performance of models for new inputs [3].

Regularizers allow us to apply penalties on layer parameters or layer activity during optimization. These penalties are summed into the loss function that the network optimizes.

These layers expose three keyword arguments:

- kernel_regularizer: Regularizer to apply a penalty on the layer's kernel
- bias_regularizer: Regularizer to apply a penalty on the layer's bias
- activity_regularizer: Regularizer to apply a penalty on the layer's output

The following built-in regularizers are available as part of the tf.keras.regularizers module (https://www.tensorflow.org/api_docs/python/tf/keras/regularizers, accessed on 05 July 2022):

L1 class

A regularizer that applies a L1 regularization penalty. The L1 regularization penalty is computed as:

- L1 class:
  A regularizer that applies an L1 regularization penalty. The L1 regularization penalty is computed as:
  $$loss = l1 \cdot reduce\_sum(abs(x)), \quad \text{(S14)}$$
  In our case, the default value used was $L1 = 0.01$.
- L2 class:
  A regularizer that applies an L2 regularization penalty. The L2 regularization penalty is computed as:
  $$loss = l2 \cdot reduce\_sum(square(x)), \quad \text{(S15)}$$
  In our case, the default value used was $L2 = 0.01$.
- L1L2 class:
  A regularizer that applies both L1 and L2 regularization penalties.
  In our case, the default values used were $L1 = 0.01$ and $L2 = 0.01$.

S5. Activation functions

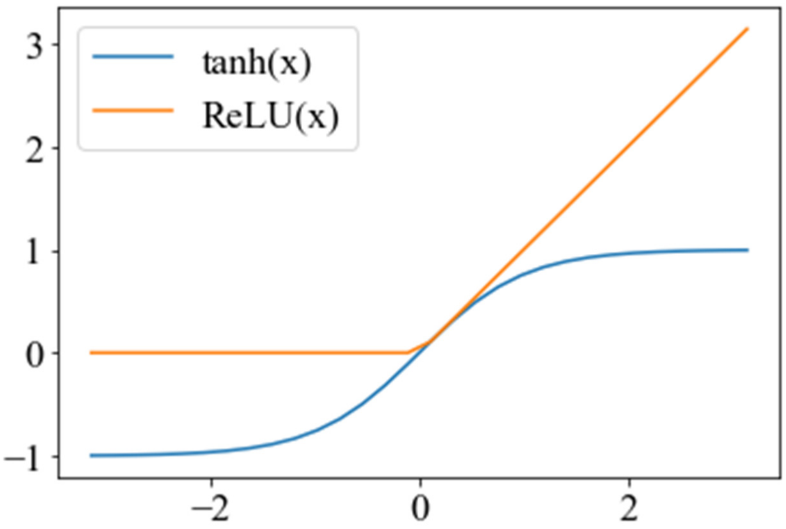

Figure S4. Activation functions considered in the hyperparameter tuning: hyperbolic tangent and rectified linear unit.

S6. Performance vs. days predicted

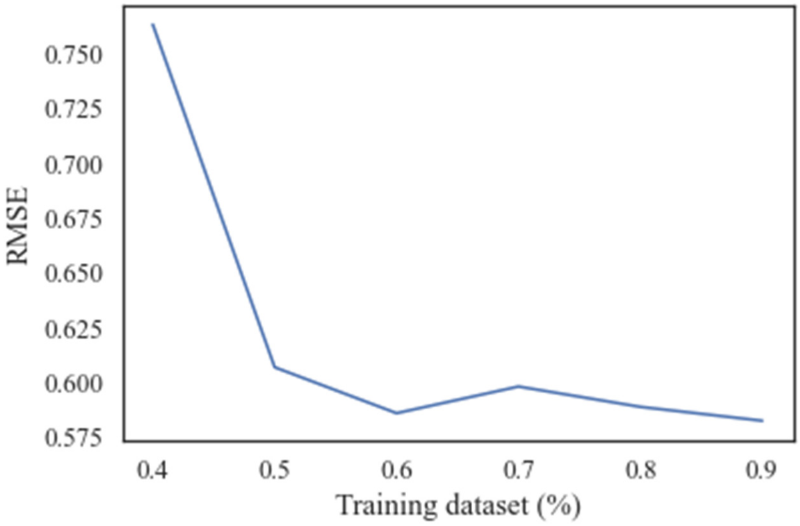

Figure S5. RMSE decreases with increasing training dataset percentage.

References

1. Ruineihart, D.E.; Hint, G.E.; Williams, R.J. *LEARNING INTERNAL REPRESENTATIONS BERROR PROPAGATION Two*; 1985;
2. Srivastava, N.; Hinton, G.; Krizhevsky, A.; Salakhutdinov, R. *Dropout: A Simple Way to Prevent Neural Networks from Overfitting*; 2014; Vol. 15;.
3. Pachitariu, M.; Sahani, M. Regularization and Nonlinearities for Neural Language Models: When Are They Needed? **2013**.
